# Fluctuations of epicardial adipose tissue and cardiovascular health: A useful biomarker? A comprehensive review

**DOI:** 10.34172/jcvtr.025.33332

**Published:** 2025-06-28

**Authors:** Bekzod Isomitdinov, Muslim Mustaev, Malikabonu Khayatova, Gentjan Jakaj, James Whiteford, Aung Ye Oo

**Affiliations:** ^1^William Harvey Research Institute, Barts and The London School of Medicine and Dentistry, Queen Mary University London, London, United Kingdom; ^2^Department of Adult Cardiac Surgery, Guy’s and St Thomas’ NHS Foundation Trust, London, United Kingdom; ^3^Department of Cardiothoracic Surgery, King’s College Hospital, London, United Kingdom; ^4^Department of Cardiothoracic Surgery, St Bartholomew’s Hospital, London, United Kingdom

**Keywords:** Epicardial adipose tissue, Epicardial fat, Pericardial adipose tissue, Coronary artery disease, Coronary atherosclerosis

## Abstract

Epicardial adipose tissue (EAT) is a fat layer of the heart located between the pericardium and myocardium and considered to be an important fat depot of the heart bearing thermoregulatory and protective functions, amongst others. Understanding of the dynamics of EAT, both positive and negative, opens new avenues for future cardiovascular research, including the development of new diagnostic and therapeutic tools. The aim of the study was to conduct a comprehensive literature review on the role of EAT, the factors influencing the change of its size, and to learn a causative relationship between fluctuations of EAT and different physiological and pathological conditions. Overall, 516 human studies indexed in PubMed, Embase, and Cochrane Library search engines (from inception up to January 2025) were screened. A total of 467 articles were excluded because they did not meet the inclusion criteria. Finally, 44 articles published from inception until January 2025 were reviewed. Our review categorises these factors into modifiable and non-modifiable, as well as aggravating and mitigating groups, to better understand their impact on EAT and cardiovascular health. Fluctuations of EAT may potentially represent a biomarker in cardiovascular research and medicine, however, it requires further validation in future studies. In such studies, it is advisable that the influencing factors are taken into consideration with adjusted normal reference ranges of EAT. Besides, the role of modifiable factors in coronary artery disease should be studied in future trials, which may shed light on the applicability of EAT as a biomarker and improve therapeutic modalities in heart disease.

## Introduction

 Epicardial adipose tissue (EAT) is a fat layer of the heart located between the pericardium and myocardium and considered to be an important fat depot of the heart bearing thermoregulatory and protectory functions, amongst others. EAT embryologically originates from splanchnopleuric mesoderm and shares blood supply with the myocardium. Therefore, it is functionally significantly different from other fat depots of the body, such as pericardial fat, abdominal fat etc.^1,2^

 Historically, the “fatty heart” has been notoriously known for its association with sudden cardiac death: in 1972 in his article, Evan Bedford quoted R. Quain’s paper from the 19^th^ century, citing that the “fatty heart” was closely associated with coronary thrombosis. Notably, the works of prominent cardiologists of the 19^th^ and 20^th^ centuries Cheyne, Stokes, Adam, Bellingham and Hayden dealt with the problems of fatty degeneration of the heart shedding light on the aetiopathogenesis of coronary artery disease and sudden death.^3^

 In the last decades, many articles have been published on the subject of EAT’s clinical value and significance, especially in various conditions such as atherosclerosis, metabolic, endocrine, oncological disorders, and physiological conditions.^4,5^

 The anatomical relationship of EAT with the myocardium and coronary arteries determines the linking and its impact on cardiometabolic profile, thus suggesting the interplay between EAT size and the above-mentioned conditions. For instance, it was shown that enlargement of EAT followed the progression of coronary artery disease (CAD) and metabolic syndrome whereas weight loss led to a reduction in EAT size.^6,7^ The current literature evidence shows a direct proportional relationship between total body fat and EAT underscoring the importance of diet and physical exercise.^8,9^ The new class of medications such as sodium-glucose co-transporter 2 (SGLT-2), glucagonlike peptide 1 (GLP-1), steroids, statins, chemotherapy agents and others have been associated with EAT size change.^10-12^ Various demographic and environmental factors such as age, sex, race, and season of the year also influence EAT size.^13^ Understanding of dynamics of EAT, both positive and negative, opens new avenues for future cardiovascular research, including the development of new diagnostic and therapeutic tools.

 The aim of the study was to conduct a comprehensive literature review of the role of EAT, the factors influencing the change of its size, and to learn a causative relationship between fluctuations of EAT and different physiological and pathological conditions.

## Methods

 In this review, comprehensive search strategies were used to identify reports of human studies indexed in PubMed, Embase, and Cochrane library search engines (from inception up to January 2025). The keywords were used to search studies relevant to our study objectives were “epicardial fat” OR “epicardial adipose tissue” AND “coronary artery disease” OR ”coronary atherosclerosis” OR “ischemic heart disease” AND “follow-up”. Moreover, the reference lists of the obtained studies were manually verified to find more related studies.

 All human studies published in English investigating the EAT thickness or volume before and after exposure of factors were included. The following study patterns were excluded: 1) not original research (reviews, editorials, non-research letters); 2) case reports or case series; 3) experimental (animal) studies; 4) conference abstracts.

 Two reviewers (BI and MM) independently screened the title and the abstracts of the obtained studies to detect potentially eligible ones. A third reviewer (AO) made the final decision about any discrepancies raised between the reviewers.

## Review

 Out of 761 articles, 245 were duplicates and were excluded. Overall, 516 studies were screened. A total of 467 articles were excluded because they did not meet the inclusion criteria. Finally, 44 articles published from inception until January 2025 and meeting the inclusion criteria were reviewed. We divided the studies into two categories by exposed factors, modifiable (medication effects, weight loss and exercise, smoking) and non-modifiable (pathological and physiological conditions, seasonal change). The reviewed articles utilised various imaging modalities (transthoracic echocardiography, computed tomography (CT) scans, positron-emission tomography-computed tomography (PET-CT) scans and cardiac magnetic resonance imaging (cMRI), therefore, the quantitative assessment of EAT in the studies was performed using the following terms: EAT size or thickness (mm), EAT volume (cm^3^), EAT density (Hounsfield units, HU), and EAT mass (g). Considering the potential discrepancy in observations due to the absence of a uniform measurement modality, this review cites the summative conclusions of the studies, emphasising the need for derived uniform thresholds of EAT change.

## Non-modifiable factors

###  Pathological and physiological conditions

####  Endocrine disorders

 In some endocrine disorders, such as Cushing syndrome, a predominant increment of the epicardial and pericardial fat was noted, which was significantly higher than in the control group (30.8 g/m^2^ vs. 17.2 g/m^2^ for EAT; 28.3 g/m^2^ vs. 11.4 g/m^2^ for pericardial adipose tissue (PAT) in one study.^14^

 On the contrary, in a number of endocrine and metabolic disorders, such as diabetes mellitus, severe obesity, and dyslipidaemia, cardiac steatosis occurs, resulting in the increased intramyocardial lipid storage along with expansion of EAT volume.^14^

 In other conditions, such as subclinical hypothyroidism, increased EAT thickness occurs due to presumably low metabolic needs and fat retention in the body. Although thyroid function test is not a reliable indicator of EAT size, low thyroid function may predispose to larger EAT thickness via general weight gain. One study observed a direct relationship between EAT and the degree of hypothyroidism: for instance, EAT thickness increased from 2.89 ± 0.38 mm on average to 4.58 ± 1.61 mm while the population group switched from subclinical hypothyroidism to overt hypothyroidism.^15^

 In the case of clinical hypothyroidism, particularly in postmenopausal women undergoing resection of thyroid cancer, EAT volume increased significantly from 147.96 cm^3^ to 166.30 cm^3^, and the authors attributed this effect to thyroid-stimulating hormone (TSH) suppression therapy.^16^

 In regards to overt hyperthyroidism, the evidence is lacking: in one study^17^ it was shown that despite normal BMI values and high levels of free T4, the mean EAT thickness was 4.31 ± 1.12 mm, which was higher than in the control group (3.11 ± 0.84 mm). Overall, the authors emphasised the association between EAT and carotid intima-media thickness with thyroid function; however, further studies on the subject are needed.

 Apart from the above-mentioned endocrine conditions, EAT change takes place in parathyroid gland disorders. For instance, according to one study,^18^ in primary hyperparathyroidism, hypercalcemia seems to play a role in increasing EAT thickness.

 Also, there is growing evidence that EAT size correlates with atherosclerosis, which is directly linked to the risk of coronary artery disease.^15^ However, one study failed to demonstrate a statistically significant relationship between EAT thickness and thyroid function test.^19^

####  Autoimmune disorders

 Some autoimmune connective tissue disorders, such as rheumatoid arthritis (RA), predispose to increased EAT volume in the presence of other cardiometabolic risk factors, mainly due to increased BMI in RA patients.^20^ Along with increased EAT volume (108.2 cm^3^ vs. 93.9 cm^3^ in the control group), patients with RA had impaired left ventricular diastolic function, as was shown in another observational study.^21^ These studies provide evidence that cardiometabolic syndrome is prevalent in RA patients as compared to individuals without RA.

 In the most recent cross-sectional study on young men with psoriasis, the mean volume of EAT was 13 cm^3^ larger than in the control group, and the coronary artery calcium score was not increased in the studied population.^22^

####  Menopause and hormone replacement therapy

 Menopausal transition contributes to endothelial ageing and dysfunction and increases EAT thickness.^23^ Hormone replacement therapy containing various doses and regimens of oestrogens also affects EAT thickness. For instance, in the KEEPS trial involving 727 menopausal women, it was shown that oral-conjugated equine oestrogens (o-CEE) may have slowed down EAT accumulation to 0.7 cm^3^ in 48 months, whereas transdermal 17b-estradiol (t-E2) augmented the association between adipose tissue accumulation and coronary artery calcification progression.^24,25^

####  Environmental and demographic factors

 In a retrospective study involving nearly 600 individuals, it was shown that a number of environmental and demographic factors, such as season, sex, and race, affect the attenuation of EAT on computed tomography, thus hindering the utilisation of EAT measurement in studies of this kind^13^. Additionally, the Rotterdam Study, which analysed a cohort of 2,524 subjects, found that gender differences exist in the distribution of cardiometabolic risk factors. For instance, the mean EAT volume of 90.1 ml was associated with increased risk in women with T2DM and obesity, whereas in men, the mean EAT volume was 121.2 ml and it was associated with increased risk of CAD.^26^

 EAT volume was shown to be closely associated with coronary atherosclerosis, obesity, and other cardiovascular risk factors. In the Japanese Shiga epidemiological study on subclinical atherosclerosis, average EAT volume significantly increased over an average interval of 4.7 years from 64.1 cm^3^ to 73.6 cm^3^ and was independently associated with smoking and heart rate during the whole period of the study.^27^ The authors suggested a strong relationship between those two factors.

## Modifiable factors

###  Medication effects

####  Hypoglycaemic therapy

 Certain hypoglycaemic medications, such as selective sodium glucose co-transporter 2 (SGLT-2) inhibitors, are associated with EAT change, according to several studies.^28-32^ For instance, in the study by Takao et al dapagliflozin significantly reduced EAT volume from 115 cm^3^ to 98.6 cm^3^ on CT scans and associated P-wave indices along with TNF-a levels.^30^ These effects may be attributed to the mechanism of reducing insulin resistance.^29^ Another study showed a beneficial effect of dapagliflozin on left ventricular systolic function in type 2 diabetes mellitus (T2DM); it also reduced EAT thickness from 7.31 ± 2.36 mm to 6.38 ± 2.03 mm in the studied population in 12 months.^31^ Another SGLT-2 inhibitor, canagliflozin, along with a reduction of blood sugar levels, reduced EAT thickness from 9.3 ± 2.5 mm to 7.3 ± 2.0 mm, which may prevent future cardiovascular events.^28^

 Another class of hypoglycaemic medications, glucagon-like peptide 1 analogue (GLP-1), liraglutide, is known to cause a substantial and rapid reduction in EAT thickness^33^ from 9.6 ± 2 mm to 6.2 ± 1.5 mm in 6 months and this effect may be linked to cardiovascular benefits in type 2 diabetic patients,^34,35^ including patients undergoing coronary artery bypass surgery.^35^

 In one non-randomised clinical trial comparing semaglutide and dulaglutide with metformin, it was shown that EAT thickness reduced on average from 9.5 ± 2.6 mm to 7.5 ± 2 mm in both GLP-1 analogues, depending on the dose of the medication.^36^ On the contrary, one prospective observational study by M. Ziyrek et al showed a significant decrease in EAT thickness from 5.07 ± 1.33 mm to 4.76 ± 1.32 mm in T2DM after monotherapy with metformin. The authors suggested that metformin may thus reduce the incidence of coronary atherosclerosis.^37^

####  Statins

 The BELLES (Beyond Endorsed Lipid Lowering with Electron Beam Tomography Scanning) trial involving 615 postmenopausal female patients with dyslipidaemia confirmed that, along with lipid-lowering effect, statins (atorvastatin and pravastatin) significantly reduced EAT by 3.38 % (EAT volume of 105 ml at baseline) at one-year follow-up.^38^ This supports a theory of the pleiotropic effect of statins by reducing metabolic activity in EAT, its cellularity and vascularity.^10,38^ In another observational study involving 195 patients with aortic stenosis, it was confirmed that atorvastatin reduced EAT thickness from 7.3 ± 0.8 mm to 6.4 ± 1.1 mm, its inflammatory status and secretory profile, supporting the pleiotropic effects of statins.^39^ Interestingly, a retrospective observational study by J. Park^40^ underlined a larger decrease in EAT thickness from 4.17 ± 1.38 mm to 3.71 ± 1.21 mm after atorvastatin therapy than with simvastatin/ezetimibe regimen.

####  Steroids 

 The role of long-term steroid therapy impacting the epicardial and pericardial fat deposition in rheumatic disorders was investigated in the observational study.^12^ It was shown that long-term (more than 6 months) steroid therapy with prednisone, both in low ( < 7.5 mg/day) and high-dose ( > 7.5 mg/day), caused accumulation of EAT and PAT from 5.7 cm^2^ to 7.2 cm^2^, more so with high-dose regimen.

####  Chemotherapy

 Some authors observed an effect of trastuzumab chemotherapy leading to EAT volume expansion in size from 114.1 ± 18.9 ml to 135.4 ± 21.2 ml and radiodensity from − 87.8 ± 2.3 HU to − 85.1 ± 2.9 HU in women undergoing treatment for breast cancer, hypothesizing that EAT may change depending on secretion of pro-inflammatory and anti-inflammatory adipokines, e.g., adiponectin, adrenomedullin, etc.^41^ On the contrary, the patients treated with anthracycline chemotherapy exhibited a reduction in EAT density from -66 HU to -71 HU due to likely myocardial necrosis and subsequent myocardial fibrosis as well as reduced myocardial metabolism, although an interpersonal variability was observed.^42^

 Another study on the influence of anthracycline chemotherapy on EAT observed an increment of the brown fat fraction of EAT from 55.75 ml to 73.31 ml, proposing a cardioprotective effect of the adipose tissue and that the EAT volume could be a new imaging marker of chemotherapy-induced cardiotoxicity and heart failure. This may be explained by the upregulation of the cardioprotective genes leading to a change in EAT.^11^ The study by Liu et al also confirmed the expansion of the EAT volume index from 3.48 ± 1.62 to 4.53 ± 1.61 mL/kg/m2 but a small, approximately 1 HU, reduction in EAT density in the patient population with breast cancer.^43^

####  Vitamins and supplements 

 The effect of vitamin and amino acid supplements has been widely investigated. For instance, in one randomised placebo-controlled trial,^44^ it was shown that the aged garlic extract with supplements, AGE-S, reduced the amount of EAT from 118 ml to 107 ml as well as pericardial, periaortic, and subcutaneous adipose tissue in 60 patients at 12 months, compared with the placebo group. This effect remained significant even after adjusting for cardiovascular risk factors and body mass index (BMI).

 Similarly, the positive effects of Omega-3 fatty acids on EAT and atherosclerosis were shown in clinical trials. In particular, the eicosapentaenoic acid (EPA) taken at a dose of 1,800 mg/day for 6 months resulted in decrements of EAT from 124 ml to 113 ml and visceral adiposity from 131 ml to 124 ml.^45^

 In the available literature, the role of leukotriene suppressors has been highlighted as well. In a multi-centre, prospective, double-blinded randomised placebo-controlled trial VIA-EAT, the 5-lypooxygenase inhibitor VIA-2291 decreased the volume of EAT by 3.0 mm^3^ and PAT by 3.0 mm^3^ in patients with recent acute coronary syndrome. However, further studies on the anti-inflammatory effect of leukotriene suppression are needed.^46^

###  Weight loss and exercise

####  Bariatric surgery

 Current evidence shows that bariatric surgery and weight loss result in a reduction of both visceral and epicardial adipose tissue. ^47-51^ One study investigated the link between the left ventricular cavity size and EAT after bariatric surgery.^50^ During the follow-up of up to 1,030 days, the authors observed an overall reduction of the left ventricular eccentricity and EAT size up to 16%, leading to reduced pericardial restraint and insulin resistance. Interestingly, the left ventricular volumes returned to the pre-operative values by the end of the follow-up period. Numerous other studies confirm these beneficial effects of bariatric surgery, including a reduction in systolic blood pressure and atherosclerosis markers such as triglycerides and cholesterol levels.^47-49^ Another study showed the association between EAT volume and weight change: the authors noted the reduction of EAT volume up to -2.3 ± 21.1% with weight loss of more than 5%, whereas weight gain led to EAT progression up to 23.3 ± 24.4%.^6^

####  Aerobic exercise

 There is a body of evidence showing that various exercise modalities reduce EAT size as well as pericardial adipose tissue and improve cardiorespiratory fitness and muscle strength.^52-56^ For instance, one study showed that both endurance and resistance exercise types increased VO_2max_, resulting in an average loss of EAT by 8 g and PAT by 15 g.^53^ In another study, the authors showed a reduction of EAT, as a result of exercise, up to -12.7 % along with loss of intrabdominal and subcutaneous adipose tissue, up to -2.4% and -1.9%, respectively.^52^ The authors of the studies^52,53^ strongly recommended that aerobic exercises be included in the treatment programs. The characteristics of the studies are shown in Table 1 and Table 2.

**Table 1:** Articles studying modifiable factors impacting EAT size before and after exposure to the influencing factor (medication effects, weight loss and exercise).

	**Study**	**Year**	**Study design**	**Study population**	**Influencing factor**	**Follow-up**	**Measurement method**	**Outcome**	**Sample size**	**Baseline**	**Change**
1.	Nakazato R^6^	2012	Cohort	Asymptomatic subjects	Weight change	4.1 ± 0.4	CT	Reduction in weight reduce EAT thickness	374	-	-
2.	Raggi P.^10^	2019	Cohort	Post-menopausal women	Atorvastatin vs. pravastatin	1 year	CT	EAT density (HU) reduced	420	-89.4 ± 24.0	-5.4 ± 29.7
3.	Kwon SS^11^	2022	Retrospective cohort	Women treated with anthracycline for breast cancer	Anthracycline	After chemotherapy.	CT	EAT volume (mm) increase	85	66.96	73.45
4.	Kitterer D^12^	2015	Prospective cohort	Long-term steroid therapy in patients with rheumatic disorders	Prednisone	6 months	Cardiac MRI	EAT volume increased cm^3^	61	5.7 [3.5–9.1]	7.2 [4.2–11.1]
5.	Wolf P^14^	2021	Cross-sectional study	Patients with Cushing syndrome	Exacerbation of disease	Before and after remission of disease	Cardiac MRI	EAT volume increased	23	-	-
6.	El Khoudary SR^24^	2019	RCT	Menopausal women	Oral conjugated equine oestrogen	48 months	CT	EAT volume (cm^3^)	474	40.6	39.9
7.	El Khoudary SR^25^	2020	RCT	Menopausal women	Oral conjugated equine estrogen	48 months	CT	EAT accumulation	467	-	-
8.	Miyazawa I^27^	2018	Observational study	Men in Japanese general population	Aging	4.7 years	CT	Pericardial fat volume increased significantly over time	623	64.1cm^3^	73.6cm^3^
9.	Yagi S^28^	2017	Observational study	T2DM	Canagliflozin	6 months	Echocardiography	EAT thickness (mm) reduced	13	9.3 ± 2.5	7.3 ± 2.0
10.	Sato T^29^	2018	RCT	T2DM and CAD	Dapagliflozin	6 months	CT	EAT volume (cm^3^) reduced	40	115 ± 22	− 16.4 ± 8.3
11.	Sato T^30^	2020	Ad-hoc of RCT	T2DM and CAD	Dapagliflozin	6 months	Echocardiography	EAT volume (cm^3^) reduced	35	113 ± 20	-15.2 ± 12.8
12.	Song X Ting^31^	2023	Observational study	T2DM	Dapagliflozin	6 months	Echocardiography	EAT thickness reduced	25	7.31 ± 2.36 mm	6.38 ± 2.03 mm
13.	Cinti^32^	2023	Clinical trial	T2DM and CAD	Dapagliflozin	4 weeks	PET/CT	EAT thickness reduced	14	0.74 ± 0.12 cm	0.60 ± 0.10 cm
14.	Iacobellis^33^	2017	Randomized, open-label, controlled study	T2DM	Liraglutide	3-6 months	Echocardiography	EAT thickness reduced	95	9.6 ± 2	6.2 ± 1.5
15.	Zhao N^34^	2021	Observational study	T2DM (with abdominal obesity and poor blood glucose control)	Liraglutide	3 months	Cardiac MRI	EAT thickness reduced	21	5.0 (5.0-7.0) mm	3:95 ± 1:43 mm
16.	Iacobellis^35^	2024	Randomized, double-blind, placebo-controlled study	T2DM and CAD	Liraglutide	12 weeks	Echocardiography	EAT thickness reduced	38	11.8 ± 2.1	-
17.	Iacobellis G^36^	2020	Clinical trial	T2DM and obesity	Semaglutide vs dulaglutide	12-week	Echocardiography	EAT thickness (mm) reduced	80	9.5 ± 2.6	7.5 ± 2
18.	Ziyrek M^37^	2019	Observational study	T2DM	Metformin	3 months	Echocardiography	EAT thickness (mm) reduced	40	5.07 ± 1.33	4.76 ± 1.32
19	Alexopoulos N^38^	2013	Subanalysis of RCT	Post-menopausal women	Atorvastatin vs. pravastatin	1 year	CT	EAT volume reduced	420	105.0 (34.9–271.6)	-3.38%
20.	Park JH^40^	2010	Retrospective cohort	Subjects after PCI	Atorvastatin vs. simvastatin/ ezetimibe	6-8 months.	Echocardiography	EAT thickness (mm) reduced	145	4.17 ± 1.38	3.71 ± 1.21
21.	Li W^41^	2022	Retrospective cohort	Women treated with trastuzumab for breast cancer	Trastuzumab	Every 3 month 4 times	Echocardiography. CT	EAT volume (ml) increased	185	112.4 ± 22.7	124.0 ± 26.1
22.	Monti CB^42^	2021	Cohort	Women treated with anthracycline for breast cancer	Anthracycline	3 months	CT	EAT density decreased	32	-66 HU	-71 HU
23.	Liu^43^	2024	Observational	Women with breast cancer	Trastuzumab Anthracycline		CT	EAT density reduced.	41	–68.20 ± 5.98	–72.55 ± 5.2
24.	Zeb I^44^	2018	RCT	Asymptomatic participants	Aged garlic extract	12 months	CT	EAT volume reduced	60	118 ± 30	-11 ± 8
25.	Sato T^45^	2014	Randomised trial	CAD patients	Eicosapentaenoic acid	6 months	CT	EAT volume (cm^3^) reduced	30	124 ± 36	113 ± 34
26.	Almeida SO^46^	2020	Post hoc of RCT	Patient post-acute coronary syndrome	Anti-inflammatory agent (VIA-2291)	24 weeks	CT	EAT volume reduced	54	-	-
27.	Kaya BC^47^	2020	Observational study	Patients undergoing bariatric surgery	Bariatric surgery	6 months	Echocardiography	EAT thickness (cm) reduced	71	0.65	0.58
28.	Salman AA^48^	2021	Case-control	Patients undergoing bariatric surgery	Bariatric surgery	12 months	Echocardiography	EAT thickness (mm) reduced	98	8.9 (1.95)	7.65 (1.67)
29.	Sorimachi H^49^	2022	Observational study	Patients undergoing bariatric surgery	Bariatric surgery	180 days	Echocardiography	EAT thickness (mm) reduced	213	7.4 ± 3.8	5.5 ± 3.5
30.	Henry JA^50^	2023	Cohort	Patients undergoing bariatric surgery	Bariatric surgery	212 days. 428 days. 1030 days.	Cardiac MRI	EAT size decrease in the early period of procedure and then return to pre-operative size.	62	-	-
31.	Henry^51^	2024	Observational	Patients undergoing bariatric surgery	Bariatric surgery	251–273- 983–1027- days	MRI	EAT volume reduced	58	-	-
32.	Kahl KG^52^	2015	RCT	Patients with depression	Exercise training	6 weeks	Cardiac MRI	EAT mass reduced	30	-	-
33.	Christensen RH^53^	2019	RCT	Physically inactive participants with abdominal obesity	Resistant exercise	12-week	Cardiac MRI	EAT mass reduced (g)	50	17	9
34.	Kim^54^	2021	Randomized controlled trial	Older women with hypertension	Taekwondo	12 weeks	CT	EAT thickness reduced	20	-	-
35.	Rosety^55^	2015	Observational	Obese women	Circuit training	12 weeks	Echocardiography	EAT thickness reduced	48	8.4 ± 1.0 mm	7.3 ± 1.3mm
36.	González^56^	2014	Randomized controlled trial	Postmenopausal women	Treadmill training	16 weeks	Echocardiography	EAT thickness (mm) reduced	60	7.3 ± 0.8	6.4 ± 1.1

**Table 2:** Articles studying non-modifiable influencing factors impacting EAT size (pathological and physiological conditions, seasonal change).

	**Study**	**Year**	**Study design**	**Influencing factor**	**Measurement method**	**Outcome**	**Sample size**	**Condition **	**Control **
1.	*Archer JM^13^	2021	Observational study	Season of the year	CT	The EAT attenuation (HU) was significantly lower during the summer than winter months	597	− 95.6 (8.0)	− 98.2 (6.9)
2.	Asik M^15^	2013	Observational study	Hypothyroidism	Echocardiography	EAT thickness (mm) higher than in control group	57	4.58 ± 1.61	2.89 ± 0.38
3.	Binnetoğlu E^17^	2014	Observational study	Hyperthyroidism	Echocardiography	EAT thickness (mm) higher than in control group	30	4.31 ± 1.12	3.11 ± 0.84
4.	Asik M^18^	2014	Observational study	Hyperparathyroidism	Echocardiography	EAT thickness (mm) higher than in control group	38	-	-
5.	Arpaci D^19^	2016	Observational study	Subclinical hypothyroidism	Echocardiography	EAT thickness (mm) higher than in control group	41	4.61 ± 0.06	4.51 ± 0.07
6.	Ormseth MJ^20^	2013	Observational study	Rheumatoid arthritis	CT	EAT volume (ml) higher than in control group	162	108.2 (77–144.6)	93.9 (69.9–133.1)
7.	Fatma E^21^	2015	Observational study	Rheumatoid arthritis	Echocardiography	EAT thickness (cm) higher than in control group	76	0.66 ± 0.20	0.54 ± 0.18
8.	Cabrera-Rego JO^23^	2018	Observational study	Post-Menopause	Echocardiography	EAT thickness (mm) higher than in control group	43	4.70 ± 1.0.	2.98 ± 1.4

*This article studied EAT density in winter (condition) and in summer (control)

## Discussion

**Figure 1:**
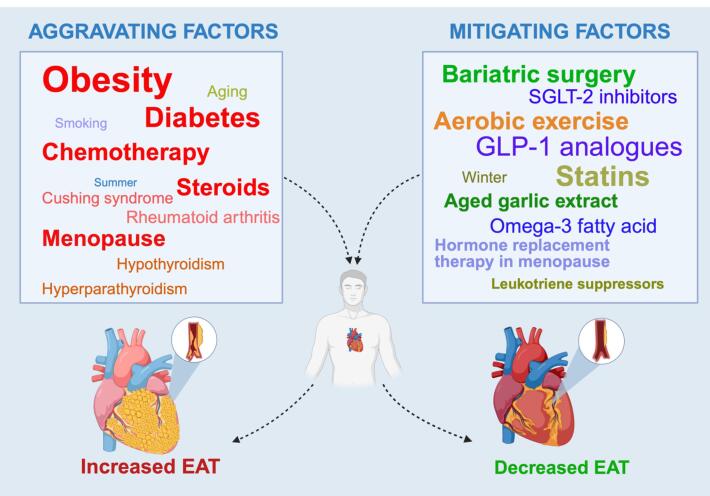
Factors influencing change of epicardial adipose tissue. Created in BioRender. Isomitdinov, B. (2025) https://BioRender.com/culombh

 Based on the available literature, EAT change can be caused by various conditions and environmental factors. Apart from the conventional division into modifiable and non-modifiable factors, we further divided them into two subsets, aggravating and mitigating factors, depending on the measured outcome. Figure 1 (Graphical abstract) represents this division of the pool of factors, attributing the effect of each according to a study’s result. For instance, obesity is a well-established aggravating factor, which was proven by a number of the analysed studies, whereas bariatric surgery bears the opposite mitigating effect.

 The weight of each factor is different depending on the depth of the scientific knowledge base. For example, about half of the included articles investigated the role of medications and medical therapy regimes, impacting EAT change. On the other hand, the less learned factors included smoking, ecological, and demographic factors.

 Importantly, EAT volume and size tend to increase in endocrine disorders such as T2DM, hypothyroidism, Cushing syndrome, and others. Also, EAT was shown to be aggravated by menopause, weight gain of different aetiology, smoking and ageing. Certain medications, such as chemotherapy agents (trastuzumab, anthracycline), and steroid therapy with prednisone, also lead to retention of EAT volume. Of note, certain connective tissue disorders, such as rheumatoid arthritis, resulted in the accumulation of EAT, however, it may well be due to concurrent steroid treatment.

 On the contrary, hypoglycaemic medications (SGLT-2 inhibitors, GLP-1 analogues, metformin) and statins (simvastatin, atorvastatin, and others) were shown to reduce EAT volume and size, thus mitigating its accumulation. Besides medications, vitamin supplements (AGE-S and omega-3) and aerobic exercise led to a reduction of EAT. Among the most investigated conditions and factors, bariatric surgery led to a significant reduction in EAT. In addition to the above-mentioned factors, seasonal changes of EAT were noticed, leading to an increase and decrease of EAT depending on the time of the year.

 Overall, the division of the known factors into aggravating and mitigating is currently observational and not exhaustive, and further studies may discriminate the role of additional factors, such as climate, geography, metabolic state and physiological responses, such as tachy- or bradycardia and others.

## Conclusion

 In summary, EAT change may represent a potential biomarker in cardiovascular research and medicine, however, it requires further validation in future studies. While at present, the utilised imaging modalities observe dynamic ranges of EAT size, thickness and volume, a common denominator in the form of “normal” reference range as well as pathological variation of EAT yet need to be determined. Following this, in future studies, the influencing factors should be taken into consideration with adjusted normal reference ranges, as these reference ranges of epicardial adipose tissue in certain conditions may be higher or lower than in other populations. The role of modifiable factors in coronary artery disease should be studied in future trials, which may shed light on the applicability of EAT as a biomarker and improve therapeutic modalities in heart disease.

 This review contributes to the current body of literature on EAT and its role in cardiovascular health. Analysing the contemporary sources from the widely used databases, it addresses the questions on the association of EAT with various pathological and physiological conditions, thus highlighting new directions for future research and potential applicability of EAT as a new biomarker of cardiovascular pathology.

 This literature review is limited to studies published in English; the review of the databases (PubMed, Embase and Cochrane Library) did not include dissertations, abstracts, editorials, case reports, conference materials and research published in other languages, which may have led to the exclusion of potentially valuable studies. The reviewed studies may be subject to publication bias and other contextual and geographical limitations. The reviewed articles did not provide cut-off points and thresholds for EAT change, which need to be investigated in future studies. The study doesn’t include a statistical analysis because of the high heterogeneity of measuring modalities and protocols.

## Competing Interests

 The authors have no conflicts of interest to declare.

## Ethical Approval

 Not Applicable.
